# Whole-Body Prepulse Inhibition Protocol to Test Sensorymotor Gating Mechanisms in Monkeys

**DOI:** 10.1371/journal.pone.0105551

**Published:** 2014-08-21

**Authors:** Patricia G. Saletti, Rafael S. Maior, Etsuro Hori, Ricardo Miyasaka de Almeida, Hisao Nishijo, Carlos Tomaz

**Affiliations:** 1 Primate Center and Laboratory of Neurosciences and Behavior, Department of Physiological Sciences, Institute of Biology, University of Brasilia, Brasilia, Brazil; 2 System Emotional Science, Graduate School of Medicine and Pharmaceutical Sciences, University of Toyama, Toyama, Japan; 3 Large Animal Teaching Hospital, Faculty of Agronomy and Veterinary Medicine, University of Brasilia, Brasilia, Brazil; Texas Christian University, United States of America

## Abstract

Prepulse inhibition (PPI) is the decrease of startle reflex amplitude when a slight stimulus is previously generated. This paradigm may provide valuable information about sensorimotor gating functionality. Here we aimed at determining the inhibited and uninhibited startle response of capuchin monkeys (*Sapajus spp*.), and to evaluate the role of the superior colliculus in PPI. Capuchin monkeys were tested in a whole-body protocol, to determine the best startle amplitude and interstimuli interval. Additionally we tested two subjects with bilateral superior colliculus damage in this protocol. Results show that 115 dB auditory pulse has induced the best startle response. In contrast to reports in other species, no habituation to the auditory stimuli was observed here in capuchins. Also, startle reflex inhibition was optimal after 120 msec interstimuli interval. Finally, there was a downward tendency of percentage inhibition in superior colliculus-lesioned monkeys. Our data provides the possibility of further studies with whole-body protocol in capuchin monkeys and reinforces the importance of the superior colliculus in PPI.

## Introduction

Acoustic startle reflex (ASR) is an innate response that causes a rapid contraction of facial and bodily muscles provoked by an intense and sharp noise [1], [2]. ASR can be used as an experimental model for the study of sensitization, habituation, prepulse inhibition, fear potentiated startle, as well as to test the effects of drugs on behaviour [3]–[6]. Prepulse inhibition (PPI) paradigm is the decrease of ASR when a slight acoustic stimulus is previously generated [2], [7], [8]. PPI depends on some stimuli-related variables, chiefly: intensity, duration, and interstimuli interval. This behavioural paradigm may provide valuable information about sensorimotor gating functionality. Understanding the neural circuitry underlying PPI may provide important insights into neurological disorders, such as schizophrenia [9]. Indeed, PPI test is generally used as a screening procedure for substances with potential antipsychotic effects [10], [11]. In this sense, tests in nonhuman primates are crucial since their results may be more readily extrapolated to humans due to brain and behavioural similarities between monkeys and human beings. Some PPI paradigm-studies have identified interstimuli intervals, as well as stimuli intensities and duration for some nonhuman primates [5], [6], [12].

Midbrain is essential for PPI [3], [13]. In an experiment with rats Yeomans *et al* [13] suggested that the mesencephalic pathways are involved in acoustic PPI. They found that a fast auditory pathway from cochlear nucleus starting from inferior colliculus to the intercollicular nucleus, then it reaches intermediate layers of the superior colliculus (SC) and proceeds to tegmental pedunculopontine nucleus, which in turn projects to the basal ganglia and into the spinal cord. Yeomans *et al* [13] also emphasized a slower multisensory pathway started in SC intermediate layers. These results support previous studies that demonstrated the importance of such structures in PPI [3], [14]. Although SC is involved in responses to visual stimuli, including detection of salient stimuli, head and eyes orientation, saccadic movements and shifts of attention [15]–[20], this subcortical structure is strongly implicated in defensive behaviours [21]–[24]. In addiction, SC receives auditory inputs from inferior colliculus [25] that must be related to PPI [13]. Therefore, SC is an important center of multisensory integration that receives visual, auditory, and somatosensory information from several cortical and subcortical areas [26]–[28].

The present study aimed to determine the pattern of startle response of individuals of *Sapajus spp*. using whole-body protocol, to determine the best intensity and PPI interstimuli interval as well as to evaluate the role of the SC in capuchin monkeys in PPI.

## Materials and Methods

### Ethics Statement

All procedures involving animals had been conducted according to guidelines of the Brazilian Society of Animal Experimentation (COBEA) and followed the Principles of Laboratory Animal Care (NIH publication no. 85-23, revised 1996). This study was approved by the Animal Ethics Committee of the Institute of Biology, University of Brasilia (UnBDOC no 128181/2011). All experiments were conducted at Primate Center at University of Brasilia, Brazil.

Animals were pair-housed in cages with natural substrate, with rope swings and nest boxes, measuring 3×3×1.8 m, under natural conditions of lightness and temperature. Animals were given access to food and water *ad libitum*. New supply of food was offered twice daily, early morning and late in the day, and water was offered by automatic drinking fountain. Animals were under constant environmental enriching tasks as usual in our Primate Center. No animal was submitted to any kind of suffering. In order to minimize stress during experiments, animals were submitted to acclimation sessions (see below), in which they received fruits reward for quiet standing in test equipment. Since animals are still under behavioural studies, no individual has been euthanized after the present study.

### Subjects

A total of 12 capuchin monkeys (*Sapajus spp.*), 8 females and 4 males, weighing between 2 and 5 kg were employed in this study. Animals were submitted to test sessions after feed time between 8–12 am, five days a week.

Subjects were placed inside the experimental chamber (see description below) by an experimenter and were submitted to 5–8 sessions for acclimation. Animals remained for 60 min per day inside the chamber with 65 dB white noise on. During this period the monkeys received fruits reward for quiet standing. Similar procedure was used for 10 min before every test session.

### Procedures

Three different tests were performed in the following sequence: startle response amplitude, prepulse inhibition and superior colliculus lesion. In startle response amplitude test, 6 animals (4 females and 2 males) were employed. In prepulse inhibition test, all the same 6 subjects were employed and we added 2 females. The 8 subjects used in prepulse inhibition test were also employed in the SC-lesion test as a control group. Beyond these 8 subjects, we employed 2 females as the sham-group and 2 males as the lesion-group. All those tests are detailed below at next sections.

**Figure 1:**
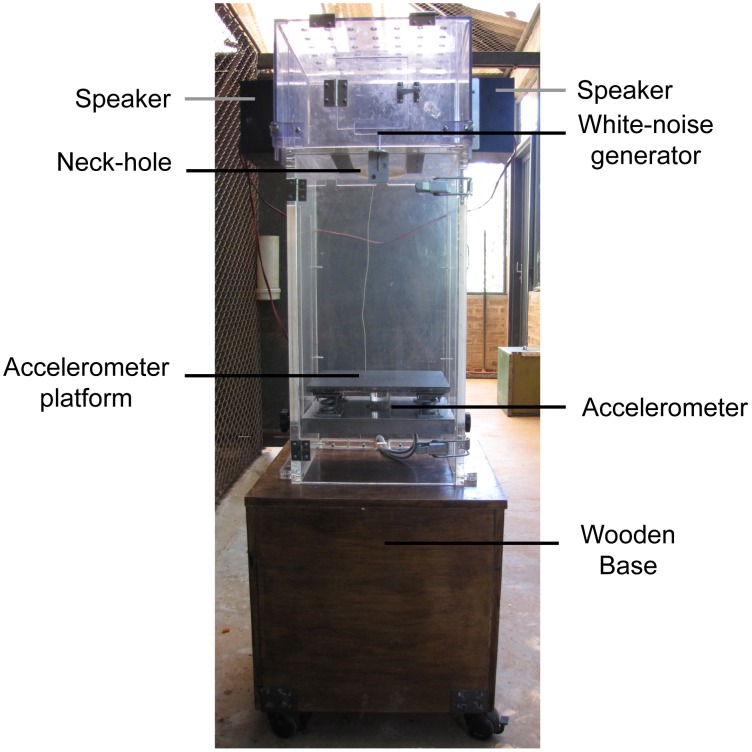
Primate test chamber. Monkeys were positioned with the neck at the neck-hole in a standing position on the accelerometer platform.

Tests were conducted in a room next to the house cage inside an acoustically isolated compartment (140×100×170 cm), with a permanent white noise of approximately 65 dB. A bottomless chamber measuring 60×30×30 cm was built in transparent acrylic material of 15 mm thick and placed above a wooden box measuring 45×40×40 cm (Fig. 1). This wooden box was built to keep the animal away from the ground improving the welfare of the subjects during tests. Subjects were placed inside the acrylic chamber with its head out. The top of the chamber had an adjustable hole that held the subjects neck. A device (30×30×25 cm) equipped with two speakers (Foster Model FT96H Frequency band; 4 KHz∼30 KHz) was placed above subject's head, whereas each speaker was 10 cm away from the head. Speakers were connected to a sound generator (O'Hara & Co., Ltd., Tokyo) and a video camera (Model Clone #1004124), which allowed continuous monitoring of the subject by an external computer during the procedure. Subject's whole-body movement were captured by an accelerometer (Inntechno Japan Co.ltd., Model: BDK3) located on the bottom of chamber and connected to an amplifier (O'Hara & Co., Ltd.). The whole system was connected to a recording software (Animal Startle – PCI 6024E, developed by O'Hara & Co., Ltd.), interfaced with Windows XP operational system, which allowed adjustment of some recording features.

### Startle response amplitude

Six animals (two males and four females) were employed in this experiment. The startle amplitude was measured in a single session with 10 equal and consecutive blocks of 5 pseudorandom white noise stimuli each (90, 95, 100, 115, 120 dB). Inter-stimulus interval was 60 sec and the duration of each stimulus was 40 msec. Startle response was defined as the maximum peak voltage amplitude of the accelerometer over 400 msec after stimulus presentation. Basal activity was defined as the maximum peak voltage over 400 msec before stimulus presentation.

### Prepulse inhibition

Eight animals (two males and six females) were employed on a single session of 7 equal and consecutive blocks of 7 pseudorandom trials each (pulse-alone, prepulse-alone and 5 prepulse-pulse trials ranging inter-stimulus intervals: 45, 70, 120, 520, 1020 msec). Pulse and prepulse were 115 dB and 80 dB white noise stimuli respectively and inter-trial interval was 60 sec. Startle response was recorded over 1800 msec after each presentation.

### Superior colliculus lesion

PPI was conducted in two males submitted to bilateral neurotoxic lesions of the SC and two females that were submitted to SC-sham surgery. Ibotenic acid (IBO) was infused in four injection sites, two in each SC (10 mg/kg in phosphate-buffered saline (PBS); Sigma-Aldrich, St. Louis, MO, USA) at a rate of 1 µl/5 min not to cause mechanical damages on those structures. In each site 0.4–0.8 µl IBO was delivered and the glass pipette was left inside the brain for 2 min to allow the dispersion and to avoid reflux during removal. Injection sites were determined by means of a stereotaxic atlas for *Sapajus* [29]. The SC-sham group was submitted to the same overall procedure, but instead IBO, the same volume of PBS vehicle was infused. For more details about surgery and evaluation of extent of injury, see Maior *et al* [23]. We also submitted the same eight animals (control group) cited above to the PPI test described in this session. Animals were employed on a single session of 10 equal and consecutive blocks of 3 pseudorandom stimuli each (pulse-alone, 115 dB, 40 msec duration; prepulse-alone, 80 dB, 20 msec duration and pulse-prepulse, 120 msec interval). Startle response was recorded as maximum peak amplitude over 600 msec after each presentation.

### Statistical Analysis

#### Startle response amplitude

One-factor repeated-measures analysis of variance (ANOVA) and *post hoc* Bonferroni test were performed to examine the effects of stimulus intensity on startle amplitude relative to basal activity, the startle response to different stimulus intensities and the mean startle response for all intensities along trial blocks.

#### Prepulse inhibition

The percent inhibition of the startle response was calculated for each subject by the following formula: 100× ([pulse-alone] - [prepulse-pulse])/(pulse-alone) as done in previous PPI studies [5], [6]. One-factor ANOVA was performed using prepulse interval and trial block as within-subjects factors. Also a *post hoc* Bonferroni test was performed to examine individual main and interaction effects.

#### Superior colliculus lesion

The details of the lesion were published elsewhere [23]. IBO injections resulted in circumscribed bilateral lesions, characterized by cell loss filled with gliosis, identified as hypersignal in T-2weighed and FLAIR scans (2-mm thick images). The lesions encompassed all SC layers down to inferior colliculus and central gray and extended two-thirds of the rostrocaudal SC axis in all subjects. No discernible damage was found in the tissues above SC due to IBO leaking during cannula retraction. Percent inhibition was calculated as explained in session 2.6.2. A Kruskal-Wallis analysis comparing the means for each group was performed to compare the percentage inhibition of the startle response among lesion, sham-lesion and control groups.

## Results

Raw data of the three tests (startle response amplitude, prepulse inhibition and superior colliculus lesion) are available in table S1, table S2 and table S3 respectively.

### Startle response amplitude

**Figure 2:**
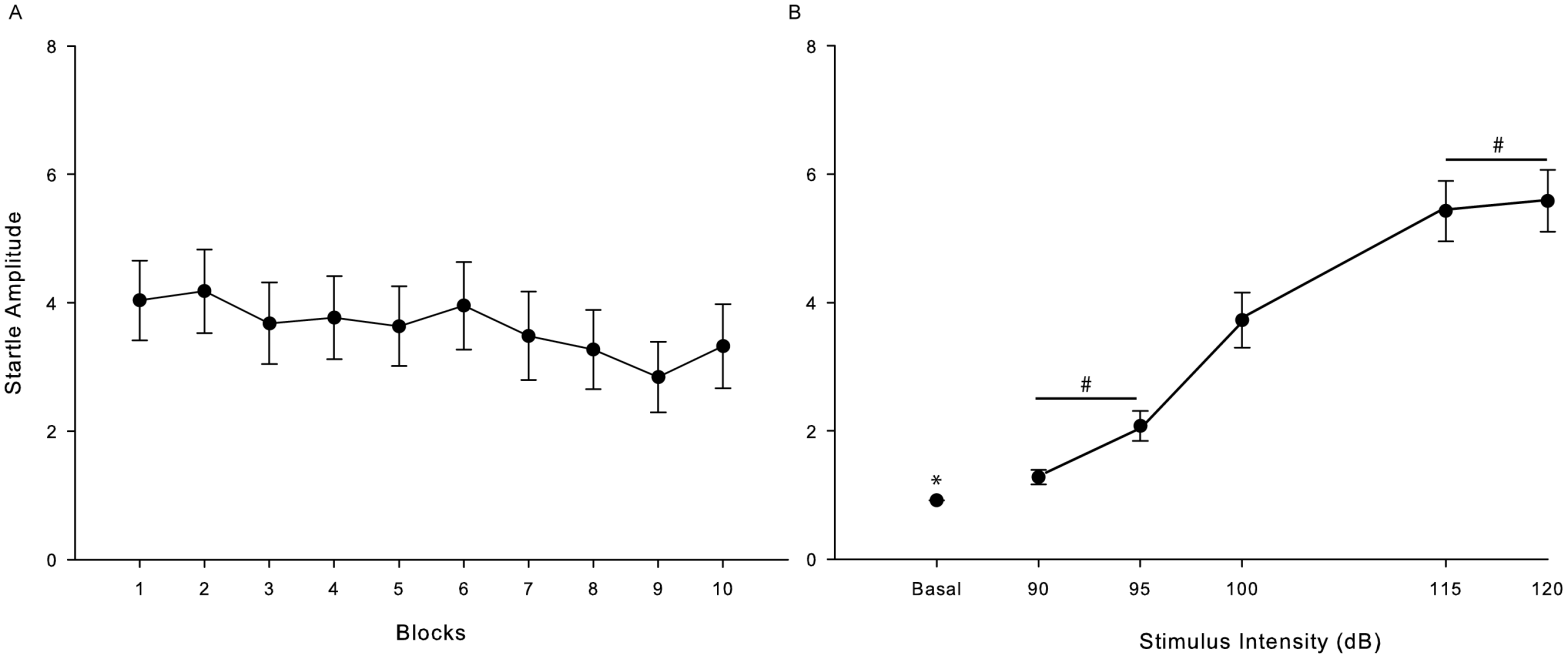
Monkeys startle response amplitude. A – Mean relative startle responses, collapsed across stimulus intensities, across repeated blocks of test trials. B - Mean relative startle responses across repeated blocks. * basal activity vs. all acoustic intensities (90–120 dB); # no statistical difference. (n = 6).

Acoustic startle response amplitude showed a gradual increase with increasing of stimulus intensity in the control monkeys. No difference was found in basal activity [*F* (0.81); p = 0.51]. There was no statistical difference over the course of 10 repeated trial blocks, showing that there was no habituation during the test trial [*F* (0.862); p = 0.55] (Fig. 2-A). Figure 2-B shows the mean amplitude startle response relative to baseline activity. All intensities caused greater startle responses than the basal activity (p<0.05). ANOVA followed by *post hoc* Bonferroni test indicated that only 90×95 dB and 115×120 dB intensities had no significant difference between themselves (p>0.05).

### Prepulse inhibition

**Figure 3:**
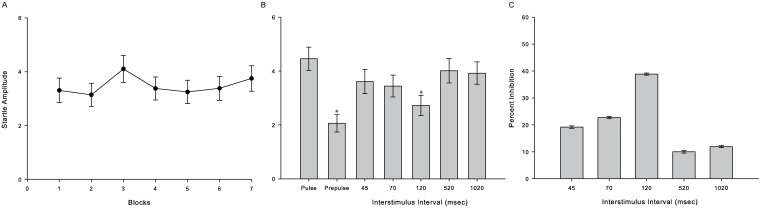
Monkeys startle response with and without prepulse stimuli. A - Mean relative startle responses, collapsed across interstimulus intervals, across 7 blocks of test trials. B - Mean relative startle responses in each test situation. * difference of pulse-alone response (p<0.05). C – Mean relative percent of startle inhibition provoked by each interval between prepulse and pulse stimuli. (n = 8).

There was no habituation over the course of 7 trial blocks in the control monkeys [*F* (0.99); p = 0.43] (Fig. 3-A). ANOVA followed by post hoc Bonferroni test indicated that only in prepulse-pulse 120 msec-interval trial mean amplitude was different of pulse-alone trial amplitude (p = 0.01). In all others cases, prepulse-pulse trials had the same response amplitude as pulse-alone trials (p>0.17). Also, prepulse-alone was different of pulse-alone response (p<0.01) (Fig. 3-B). Figure 3-C shows percentage of inhibition of startle response of each interstimulus interval tested.

### Superior colliculus lesions

**Figure 4:**
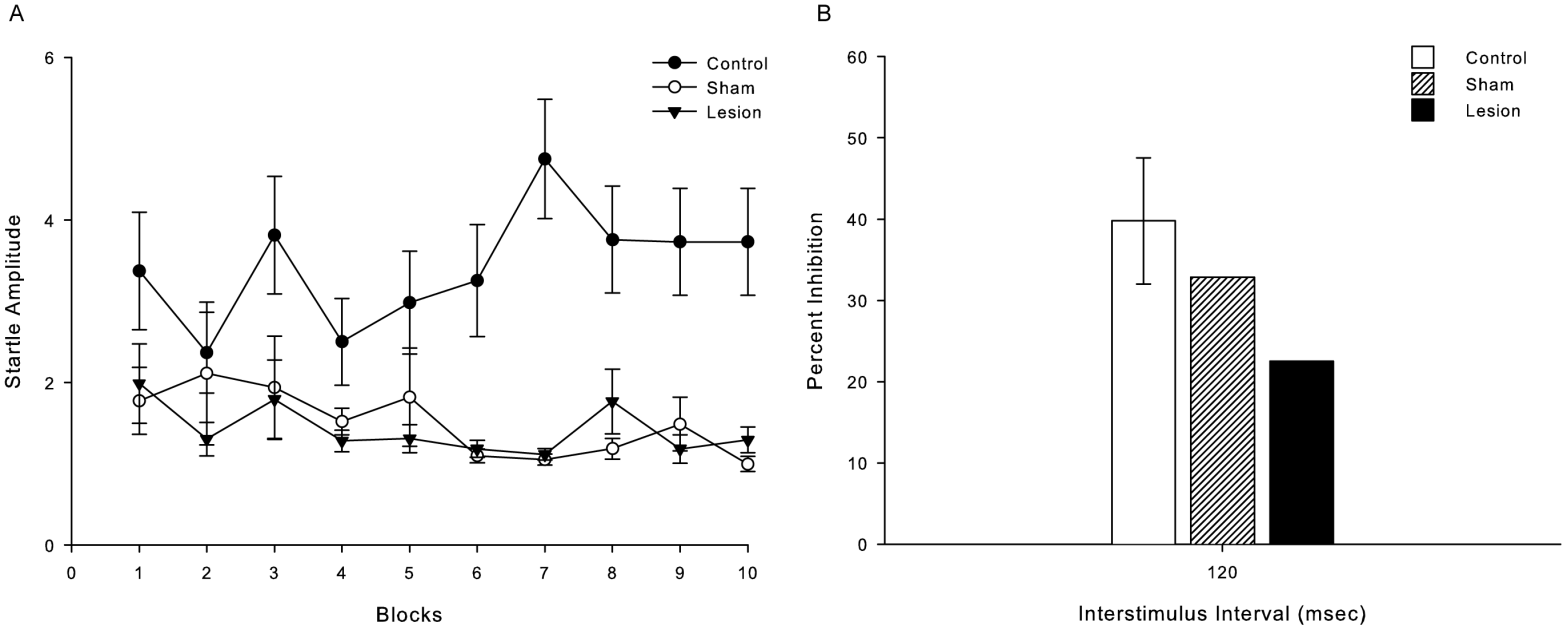
SC-lesion, sham and control monkeys startle response. A – Mean relative startle responses, collapsed across the three test trials, across 10 blocks. B – Mean relative percent inhibition of startle response in control (n = 8), sham (n = 2) and lesion (n = 2) groups in prepulse-pulse 120 msec-interval protocol.

There was no habituation over the course of 10 trial blocks for any of the groups [control: *F* (1.11); p = 0.15, sham: *F* (0.86); p = 0.57, lesion: *F* (1.19); p = 0.31] (Fig. 4-A). As seen in Fig. 4-B, Kruskal-Wallis analyses yielded no significant difference on percent inhibition between the three groups despite a tendency to deficit of prepulse inhibition in SC-lesion animals (x^2^ = 2.965; p = 0.227).

## Discussion

In the present study, we demonstrated startle response in *Sapajus spp.* in a whole-body paradigm. Our findings are consistent with the study accomplished by Winslow *et al*. [5] in rhesus monkeys, which revealed the possibility to evaluate *Macaca* startle pattern with the measurement of whole-body activity. Now we demonstrated that with the same protocol, capuchin and rhesus monkeys exhibit the same pattern of startle and PPI responses. To our knowledge there is only one previous report of PPI in capuchin monkeys. Linn and Javitt [12] adapted an eyeblink protocol apparatus for testing in humans and found similar results as the present study.

The pattern of PPI response followed expectations. We found that 115 dB and 120 dB were equally good to cause startle response. Considering that 115 dB was capable of inducing the expected response in our subjects and that it is also the same intensity found in literature for other primates [5], [6], [12], [30], this intensity was selected as startle intensity for the subsequent tests. As in other nonhuman primate species, a prepulse intensity of 80 dB was ideal to inhibit the startle response generated by an 115 dB pulse. Also, the interstimuli interval that best inhibited startle response was 120 msec, in keeping with previous reports in other nonhuman primates [5], [6], [12], [30].

In the SC-lesion test, we intended to evaluate our experimental protocol and test the role of SC on PPI response in primates. Thus, it was possible to show that there is a downward tendency of prepulse inhibition in animals with SC damage as well as demonstrated in rats [14], [31]. Although caution should be taken when analysing this tentative study, the percentage inhibition found was not significant across the experimental groups. SC is an important and well-known structure related to visual information (for review [22]), however SC also receives multisensory information, such as tactile and auditory [26], [32], [33]. SC deep layers are also related to defensive behaviours such as freezing, darting and shift of attention in rodents [20], [34], [35], as well as prey-predator behaviour in primates [23], [24]. Therefore, further testing might lead to a better understanding of SC's role in nonhuman primate PPI.

Habituation is a learning process whereby behavioural responses decrease after repeated stimulations [36], [37]. As seen in Figs. 2-A and 3-A, we observe a non-significant reduction of the startle amplitude during the session test, i.e., no habituation was observed. In humans, 13 trials are optimal to reduce behavioural responses with a 100 dB startle stimulus [38] and in rhesus monkeys a small decrease in startle amplitude has been observed after 5 blocks of stimuli presentation within session [5]. Interestingly, deficits in startle habituation have been observed in schizophrenic patients [39], [40]. Habituation in subjects with damage to the superior colliculus was also not observed (Fig. 4-A). Nevertheless, it is known that schizophrenia patients show deficits in habituation due to dysfunction on the hippocampus, which indicates that this reduction of behavioural response may be correlated with memory performance [41] and perhaps there is no relation with the superior colliculus. Regardless, the lack of habituation in observed here suggests that PPI testing on this species may help understand the habituation patterns seen in schizophrenic patients.

The validation of the PPI test with whole-body experimental protocol using capuchin monkeys enables new preclinical studies to test potential antipsychotic substances. Many studies have been conducted with rodents [42]–[44], but due to *Sapajus* phylogenetic proximity with humans, the use of these animals as experimental models for drug testing may yield more reliable results.

## Conclusions

The present study validates the PPI paradigm for testing in capuchin monkeys. Habituation to auditory stimuli was not seen here in *Sapajus* as reported in humans and rhesus. This was the first study that shows PPI responses of SC-lesioned primates, thus the results might be relevant to understand the role of the SC in humans' neurological disorders with impairment in sensorimotor gating mechanisms, as schizophrenia. Further validation of our experimental protocol enables future studies in order to find new antipsychotic drugs.

## Supporting Information

Checklist S1 **ARRIVE guidelines checklist.**
(PDF)

Table S1 **Startle response of animals in** ***Startle response amplitude*** **test.**
(PDF)

Table S2 **Startle response of animals in** ***Prepulse inhibition*** **test.**
(PDF)

Table S3 **Startle response of animals in** ***Superior colliculus lesions*** **test.**
(PDF)
