# Supplementary material for: Whole-Body Prepulse Inhibition Protocol to Test Sensorymotor Gating Mechanisms in Monkeys
Source: PLoS One. 2014 Aug 21;9(8):e105551. doi: 10.1371/journal.pone.0105551 (PMC4140807; doi:10.1371/journal.pone.0105551)
Supplement: Table S1 — Startle response of animals in Startle response amplitude test. (PDF) [file pone.0105551.s002.pdf]

Table S1.Startle response of animals in Startle response amplitude test.

Subject	Block	Trial	db	PrePtoP	PtoP
F01	0	0	90	0,771	0,747
F01	1	0	90	0,669	0,747
F01	2	0	90	0,728	0,732
F01	3	0	90	0,693	0,664
F01	4	0	90	0,762	0,845
F01	5	0	90	0,864	0,757
F01	6	0	90	0,703	0,654
F01	7	0	90	0,776	0,82
F01	8	0	90	0,796	0,679
F01	9	0	90	0,723	0,845
F02	0	0	90	0,801	5,625
F02	1	0	90	0,933	5,459
F02	2	0	90	0,791	4,6
F02	3	0	90	0,659	2,373
F02	4	0	90	0,63	1,899
F02	5	0	90	0,742	1,562
F02	6	0	90	1,021	5,742
F02	7	0	90	0,732	1,172
F02	8	0	90	0,83	4,531
F02	9	0	90	0,786	1,343
F03	0	0	90	0,732	1,558
F03	1	0	90	0,747	1,03
F03	2	0	90	0,659	3,032
F03	3	0	90	0,64	4,404
F03	4	0	90	0,64	1,299
F03	5	0	90	0,811	2,69
F03	6	0	90	0,713	0,737
F03	7	0	90	0,688	0,835
F03	8	0	90	0,737	0,718
F03	9	0	90	0,771	0,781
F04	0	0	90	0,977	0,884
F04	1	0	90	0,923	0,874
F04	2	0	90	0,703	1,021
F04	3	0	90	0,918	0,869
F04	4	0	90	0,874	0,898
F04	5	0	90	0,962	1,289
F04	6	0	90	0,894	0,947
F04	7	0	90	0,879	0,933
F04	8	0	90	0,801	0,913
F04	9	0	90	1,045	0,889
M01	0	0	90	1,06	0,942
M01	1	0	90	0,986	1,011
M01	2	0	90	1,011	1,045
M01	3	0	90	1,162	0,981
M01	4	0	90	0,991	1,187
M01	5	0	90	1,045	1,064
M01	6	0	90	1,152	1,206
M01	7	0	90	1,201	1,333
M01	8	0	90	0,898	1,47
M01	9	0	90	1,04	1,147

M02	0	0	90	1,055	4,15
M02	1	0	90	1,182	2,207
M02	2	0	90	1,094	1,992
M02	3	0	90	1,089	1,196
M02	4	0	90	1,177	1,162
M02	5	0	90	1,108	1,719
M02	6	0	90	1,05	2,363
M02	7	0	90	1,201	1,279
M02	8	0	90	1,001	1,152
M02	9	0	90	1,157	1,152
F01	0	1	95	0,894	0,737
F01	1	1	95	0,825	1,196
F01	2	1	95	0,713	0,723
F01	3	1	95	0,835	0,894
F01	4	1	95	0,894	0,757
F01	5	1	95	0,645	0,889
F01	6	1	95	0,835	0,742
F01	7	1	95	0,742	0,625
F01	8	1	95	0,723	0,796
F01	9	1	95	0,684	0,82
F02	0	1	95	0,762	4,473
F02	1	1	95	0,718	6,777
F02	2	1	95	1,06	2,969
F02	3	1	95	0,723	2,983
F02	4	1	95	0,659	1,318
F02	5	1	95	0,703	2,168
F02	6	1	95	3,145	3,179
F02	7	1	95	0,645	1,646
F02	8	1	95	0,684	5,205
F02	9	1	95	0,635	0,879
F03	0	1	95	0,723	3,13
F03	1	1	95	0,811	4,741
F03	2	1	95	0,791	1,958
F03	3	1	95	0,698	2,759
F03	4	1	95	0,684	2,9
F03	5	1	95	0,649	1,714
F03	6	1	95	0,776	1,416
F03	7	1	95	0,649	0,889
F03	8	1	95	0,752	4,536
F03	9	1	95	0,84	2,988
F04	0	1	95	0,859	0,874
F04	1	1	95	0,815	1,299
F04	2	1	95	0,859	0,869
F04	3	1	95	0,884	1,104
F04	4	1	95	0,854	2,207
F04	5	1	95	0,879	1,006
F04	6	1	95	0,85	1,724
F04	7	1	95	0,835	0,952
F04	8	1	95	0,874	1,03
F04	9	1	95	0,981	0,894
M01	0	1	95	0,952	0,996
M01	1	1	95	0,991	1,548
M01	2	1	95	0,913	1,401

M01	3	1	95	1,035	1,47
M01	4	1	95	0,991	1,05
M01	5	1	95	1,016	1,265
M01	6	1	95	0,913	2,891
M01	7	1	95	0,996	2,036
M01	8	1	95	0,981	1,343
M01	9	1	95	1,182	1,152
M02	0	1	95	1,211	6,211
M02	1	1	95	1,157	7,949
M02	2	1	95	1,187	6,167
M02	3	1	95	1,182	2,715
M02	4	1	95	1,104	1,46
M02	5	1	95	1,357	1,514
M02	6	1	95	1,289	4,507
M02	7	1	95	1,099	4,409
M02	8	1	95	1,011	3,491
M02	9	1	95	1,055	3,096
F01	0	2	100	0,669	1,631
F01	1	2	100	0,728	0,898
F01	2	2	100	0,815	1,704
F01	3	2	100	0,942	1,865
F01	4	2	100	0,781	0,747
F01	5	2	100	0,742	0,806
F01	6	2	100	0,84	0,84
F01	7	2	100	0,747	0,864
F01	8	2	100	0,737	0,732
F01	9	2	100	0,747	0,664
F02	0	2	100	0,723	5,542
F02	1	2	100	1,279	7,139
F02	2	2	100	0,757	7,495
F02	3	2	100	0,874	1,87
F02	4	2	100	0,869	2,183
F02	5	2	100	1,23	3,159
F02	6	2	100	0,732	5,044
F02	7	2	100	2,754	2,695
F02	8	2	100	0,771	2,764
F02	9	2	100	1,953	2,446
F03	0	2	100	0,752	2,134
F03	1	2	100	0,674	6,626
F03	2	2	100	0,664	5,845
F03	3	2	100	0,752	9,58
F03	4	2	100	0,742	9,16
F03	5	2	100	0,82	5,317
F03	6	2	100	0,752	2,061
F03	7	2	100	0,664	4,697
F03	8	2	100	0,752	7,827
F03	9	2	100	0,747	8,447
F04	0	2	100	0,84	4,16
F04	1	2	100	0,786	6,006
F04	2	2	100	0,894	1,182
F04	3	2	100	1,245	2,881
F04	4	2	100	0,757	2,671
F04	5	2	100	0,815	3,853

F04	6	2	100	0,962	0,884
F04	7	2	100	0,854	1,206
F04	8	2	100	0,957	0,806
F04	9	2	100	1,025	0,864
M01	0	2	100	1,025	2,49
M01	1	2	100	0,947	2,749
M01	2	2	100	1,021	1,743
M01	3	2	100	1,006	2,354
M01	4	2	100	0,923	1,67
M01	5	2	100	1,206	2,905
M01	6	2	100	0,972	2,285
M01	7	2	100	1,021	1,89
M01	8	2	100	1,011	1,538
M01	9	2	100	1,016	1,157
M02	0	2	100	0,991	4,736
M02	1	2	100	1,313	7,339
M02	2	2	100	1,152	4,175
M02	3	2	100	1,035	9,209
M02	4	2	100	1,162	4,946
M02	5	2	100	1,147	10,459
M02	6	2	100	1,372	9,39
M02	7	2	100	1,675	8,74
M02	8	2	100	1,182	1,055
M02	9	2	100	1,167	8,481
F01	0	3	115	0,85	4,946
F01	1	3	115	0,669	6,655
F01	2	3	115	0,791	5,474
F01	3	3	115	0,708	2,026
F01	4	3	115	0,781	8,413
F01	5	3	115	0,63	3,408
F01	6	3	115	0,708	0,962
F01	7	3	115	0,723	2,856
F01	8	3	115	0,708	0,742
F01	9	3	115	0,601	3,271
F02	0	3	115	0,835	9,614
F02	1	3	115	0,83	5,278
F02	2	3	115	1,074	8,779
F02	3	3	115	0,786	10,01
F02	4	3	115	0,884	8,584
F02	5	3	115	1,836	3,667
F02	6	3	115	1,484	2,212
F02	7	3	115	0,562	3,271
F02	8	3	115	1,206	3,428
F02	9	3	115	0,786	9,922
F03	0	3	115	0,693	9,419
F03	1	3	115	0,718	8,818
F03	2	3	115	0,732	8,696
F03	3	3	115	0,703	11,157
F03	4	3	115	0,781	9,531
F03	5	3	115	0,645	10,957
F03	6	3	115	0,728	11,377
F03	7	3	115	0,625	9,233
F03	8	3	115	0,62	7,104

F03	9	3	115	0,688	9,004
F04	0	3	115	0,918	8,271
F04	1	3	115	0,933	1,177
F04	2	3	115	0,728	2,256
F04	3	3	115	0,757	3,779
F04	4	3	115	0,796	2,397
F04	5	3	115	0,732	3,101
F04	6	3	115	0,889	4,102
F04	7	3	115	1,011	3,228
F04	8	3	115	0,908	4,629
F04	9	3	115	0,864	1,646
M01	0	3	115	1,123	1,675
M01	1	3	115	1,03	3,984
M01	2	3	115	0,986	2,695
M01	3	3	115	1,064	3,755
M01	4	3	115	1,021	4,927
M01	5	3	115	0,913	3,418
M01	6	3	115	1,26	3,257
M01	7	3	115	1,001	2,261
M01	8	3	115	0,937	2,134
M01	9	3	115	1,025	1,299
M02	0	3	115	1,152	4,624
M02	1	3	115	1,177	9,795
M02	2	3	115	1,064	8,984
M02	3	3	115	1,309	4,146
M02	4	3	115	1,064	3,887
M02	5	3	115	1,23	9,131
M02	6	3	115	1,23	11,421
M02	7	3	115	1,45	2,275
M02	8	3	115	1,377	8,643
M02	9	3	115	1,167	10,269
F01	0	4	120	0,674	8,433
F01	1	4	120	0,747	3,95
F01	2	4	120	0,972	5,596
F01	3	4	120	0,698	3,267
F01	4	4	120	0,732	8,589
F01	5	4	120	0,679	4,199
F01	6	4	120	0,771	4,277
F01	7	4	120	0,884	2,549
F01	8	4	120	0,664	5,859
F01	9	4	120	0,791	4,062
F02	0	4	120	2,217	6,953
F02	1	4	120	0,815	9,79
F02	2	4	120	0,806	5,845
F02	3	4	120	0,811	10,347
F02	4	4	120	1,479	5,269
F02	5	4	120	0,781	9,937
F02	6	4	120	1,001	4,81
F02	7	4	120	0,791	1,807
F02	8	4	120	1,06	5,483
F02	9	4	120	0,742	4,092
F03	0	4	120	0,605	10,298
F03	1	4	120	0,786	7,187

F03	2	4	120	0,757	10,322
F03	3	4	120	0,85	9,346
F03	4	4	120	0,64	7,725
F03	5	4	120	0,708	8,77
F03	6	4	120	0,718	10,566
F03	7	4	120	0,801	9,995
F03	8	4	120	0,703	8,931
F03	9	4	120	0,713	7,246
F04	0	4	120	0,933	3,12
F04	1	4	120	0,85	3,901
F04	2	4	120	0,923	1,416
F04	3	4	120	0,869	3,398
F04	4	4	120	0,967	2,178
F04	5	4	120	0,986	7,49
F04	6	4	120	0,981	3,384
F04	7	4	120	1,011	6,133
F04	8	4	120	0,913	1,309
F04	9	4	120	0,923	1,909
M01	0	4	120	1,24	4,902
M01	1	4	120	0,952	1,499
M01	2	4	120	1,04	2,119
M01	3	4	120	1,045	1,196
M01	4	4	120	1,094	1,719
M01	5	4	120	1,079	1,313
M01	6	4	120	0,991	1,411
M01	7	4	120	1,03	1,733
M01	8	4	120	1,006	1,045
M01	9	4	120	0,923	1,772
M02	0	4	120	1,079	9,863
M02	1	4	120	1,211	11,265
M02	2	4	120	1,26	10,815
M02	3	4	120	1,094	9,214
M02	4	4	120	1,089	8,506
M02	5	4	120	1,099	9,785
M02	6	4	120	1,138	3,682
M02	7	4	120	1,177	9,932
M02	8	4	120	1,245	2,627
M02	9	4	120	1,118	9,277
