# Supplementary material for: Whole-Body Prepulse Inhibition Protocol to Test Sensorymotor Gating Mechanisms in Monkeys
Source: PLoS One. 2014 Aug 21;9(8):e105551. doi: 10.1371/journal.pone.0105551 (PMC4140807; doi:10.1371/journal.pone.0105551)
Supplement: Table S2 — Startle response of animals in Prepulse inhibition test. (PDF) [file pone.0105551.s003.pdf]

Table S2. Startle response of animals in Prepulse inhibition test.

Subject	Block	Trial	Test condition	PtoP
F05	0	0	115db	3,872
F05	1	0	115db	1,44
F05	2	0	115db	9,756
F05	3	0	115db	1,357
F05	4	0	115db	10,264
F05	5	0	115db	9,287
F05	6	0	115db	10,825
F05	7	0	115db	6,06
F06	0	0	115db	1,528
F06	1	0	115db	1,235
F06	2	0	115db	6,753
F06	3	0	115db	1,47
F06	4	0	115db	1,885
F06	5	0	115db	1,636
F06	6	0	115db	4,502
F06	7	0	115db	0,859
F02	0	0	115db	8,691
F02	1	0	115db	7,949
F02	2	0	115db	5,591
F02	3	0	115db	4,111
F02	4	0	115db	6,372
F02	5	0	115db	4,033
F02	6	0	115db	4,478
F02	7	0	115db	9,751
F01	0	0	115db	4,59
F01	1	0	115db	1,396
F01	2	0	115db	2,808
F01	3	0	115db	2,148
F01	4	0	115db	1,997
F01	5	0	115db	1,299
F01	6	0	115db	3,247
F01	7	0	115db	1,274
M01	0	0	115db	1,069
M01	1	0	115db	1,289
M01	2	0	115db	3,574
M01	3	0	115db	2,778
M01	4	0	115db	2,148
M01	5	0	115db	0,825
M01	6	0	115db	1,826
M01	7	0	115db	0,957
F03	0	0	115db	10,454
F03	1	0	115db	8,359
F03	2	0	115db	10,625
F03	3	0	115db	10,967
F03	4	0	115db	10,913
F03	5	0	115db	10,073

F03	6	0	115db	7,378
F03	7	0	115db	5,146
M02	0	0	115db	1,65
M02	1	0	115db	4,014
M02	2	0	115db	6,343
M02	3	0	115db	8,975
M02	4	0	115db	0,874
M02	5	0	115db	6,46
M02	6	0	115db	5,327
M02	7	0	115db	4,404
F04	0	0	115db	5,947
F04	1	0	115db	2,856
F04	2	0	115db	9,712
F04	3	0	115db	3,784
F04	4	0	115db	8,408
F04	5	0	115db	2,524
F04	6	0	115db	4,541
F04	7	0	115db	9,351
F05	0	1	80db	0,952
F05	1	1	80db	0,889
F05	2	1	80db	1,411
F05	3	1	80db	1,025
F05	4	1	80db	6,743
F05	5	1	80db	0,854
F05	6	1	80db	12,002
F05	7	1	80db	3,301
F06	0	1	80db	1,03
F06	1	1	80db	0,645
F06	2	1	80db	0,737
F06	3	1	80db	0,742
F06	4	1	80db	0,85
F06	5	1	80db	9,619
F06	6	1	80db	1,162
F06	7	1	80db	1,074
F02	0	1	80db	1,128
F02	1	1	80db	1,172
F02	2	1	80db	1,196
F02	3	1	80db	1,099
F02	4	1	80db	1,323
F02	5	1	80db	1,343
F02	6	1	80db	3,584
F02	7	1	80db	4,424
F01	0	1	80db	0,811
F01	1	1	80db	0,986
F01	2	1	80db	0,796
F01	3	1	80db	1,26
F01	4	1	80db	0,879
F01	5	1	80db	0,869
F01	6	1	80db	1,089

F01	7	1	80db	0,996
M01	0	1	80db	0,937
M01	1	1	80db	1,045
M01	2	1	80db	0,933
M01	3	1	80db	0,845
M01	4	1	80db	0,757
M01	5	1	80db	0,967
M01	6	1	80db	0,845
M01	7	1	80db	0,903
F03	0	1	80db	0,894
F03	1	1	80db	1,201
F03	2	1	80db	0,981
F03	3	1	80db	1,035
F03	4	1	80db	0,972
F03	5	1	80db	0,869
F03	6	1	80db	8,774
F03	7	1	80db	9,883
M02	0	1	80db	0,811
M02	1	1	80db	0,605
M02	2	1	80db	0,728
M02	3	1	80db	0,747
M02	4	1	80db	0,708
M02	5	1	80db	0,762
M02	6	1	80db	5,586
M02	7	1	80db	5,693
F04	0	1	80db	1,719
F04	1	1	80db	1,685
F04	2	1	80db	1,777
F04	3	1	80db	1,523
F04	4	1	80db	1,689
F04	5	1	80db	1,665
F04	6	1	80db	7,773
F04	7	1	80db	5,674
F05	0	2	45ISI	9,404
F05	1	2	45ISI	8,73
F05	2	2	45ISI	9,814
F05	3	2	45ISI	11,636
F05	4	2	45ISI	9,028
F05	5	2	45ISI	3,823
F05	6	2	45ISI	10,889
F05	7	2	45ISI	4,238
F06	0	2	45ISI	8,687
F06	1	2	45ISI	1,841
F06	2	2	45ISI	5,225
F06	3	2	45ISI	1,479
F06	4	2	45ISI	2,363
F06	5	2	45ISI	1,738
F06	6	2	45ISI	1,025
F06	7	2	45ISI	9,531

F02	0	2	45ISI	1,665
F02	1	2	45ISI	6,172
F02	2	2	45ISI	3,799
F02	3	2	45ISI	6,592
F02	4	2	45ISI	4,932
F02	5	2	45ISI	8,584
F02	6	2	45ISI	5,068
F02	7	2	45ISI	7,178
F01	0	2	45ISI	0,806
F01	1	2	45ISI	1,035
F01	2	2	45ISI	0,757
F01	3	2	45ISI	0,845
F01	4	2	45ISI	0,767
F01	5	2	45ISI	0,854
F01	6	2	45ISI	0,796
F01	7	2	45ISI	1,245
M01	0	2	45ISI	0,957
M01	1	2	45ISI	0,884
M01	2	2	45ISI	0,815
M01	3	2	45ISI	0,879
M01	4	2	45ISI	0,981
M01	5	2	45ISI	0,977
M01	6	2	45ISI	0,967
M01	7	2	45ISI	0,859
F03	0	2	45ISI	10,508
F03	1	2	45ISI	10,469
F03	2	2	45ISI	10,679
F03	3	2	45ISI	9,199
F03	4	2	45ISI	8,765
F03	5	2	45ISI	8,345
F03	6	2	45ISI	4,653
F03	7	2	45ISI	8,613
M02	0	2	45ISI	1,138
M02	1	2	45ISI	5,498
M02	2	2	45ISI	1,157
M02	3	2	45ISI	4,136
M02	4	2	45ISI	0,903
M02	5	2	45ISI	1,045
M02	6	2	45ISI	3,608
M02	7	2	45ISI	1,035
F04	0	2	45ISI	2,383
F04	1	2	45ISI	2,354
F04	2	2	45ISI	3,057
F04	3	2	45ISI	2,329
F04	4	2	45ISI	1,978
F04	5	2	45ISI	1,821
F04	6	2	45ISI	1,714
F04	7	2	45ISI	1,953
F05	0	3	70ISI	6,714

F05	1	3	70ISI	11,938
F05	2	3	70ISI	6,753
F05	3	3	70ISI	9,229
F05	4	3	70ISI	5,386
F05	5	3	70ISI	10,649
F05	6	3	70ISI	7,93
F05	7	3	70ISI	9,136
F06	0	3	70ISI	1,519
F06	1	3	70ISI	1,24
F06	2	3	70ISI	1,646
F06	3	3	70ISI	2,139
F06	4	3	70ISI	0,82
F06	5	3	70ISI	1,558
F06	6	3	70ISI	0,757
F06	7	3	70ISI	1,597
F02	0	3	70ISI	6,597
F02	1	3	70ISI	4,844
F02	2	3	70ISI	3,384
F02	3	3	70ISI	5,947
F02	4	3	70ISI	8,022
F02	5	3	70ISI	4,155
F02	6	3	70ISI	8,921
F02	7	3	70ISI	6,309
F01	0	3	70ISI	1,499
F01	1	3	70ISI	0,903
F01	2	3	70ISI	0,723
F01	3	3	70ISI	1,021
F01	4	3	70ISI	0,781
F01	5	3	70ISI	0,996
F01	6	3	70ISI	0,752
F01	7	3	70ISI	1,006
M01	0	3	70ISI	0,884
M01	1	3	70ISI	0,957
M01	2	3	70ISI	0,967
M01	3	3	70ISI	0,972
M01	4	3	70ISI	0,781
M01	5	3	70ISI	0,996
M01	6	3	70ISI	0,767
M01	7	3	70ISI	0,991
F03	0	3	70ISI	10,41
F03	1	3	70ISI	6,421
F03	2	3	70ISI	7,915
F03	3	3	70ISI	2,71
F03	4	3	70ISI	9,829
F03	5	3	70ISI	4,854
F03	6	3	70ISI	8,286
F03	7	3	70ISI	1,299
M02	0	3	70ISI	6,953
M02	1	3	70ISI	1,709

M02	2	3	70ISI	3,301
M02	3	3	70ISI	2,456
M02	4	3	70ISI	1,074
M02	5	3	70ISI	5,791
M02	6	3	70ISI	3,208
M02	7	3	70ISI	0,742
F04	0	3	70ISI	1,831
F04	1	3	70ISI	1,904
F04	2	3	70ISI	1,904
F04	3	3	70ISI	1,895
F04	4	3	70ISI	1,709
F04	5	3	70ISI	1,763
F04	6	3	70ISI	2,896
F04	7	3	70ISI	2,051
F05	0	4	120ISI	8,511
F05	1	4	120ISI	5,308
F05	2	4	120ISI	10,635
F05	3	4	120ISI	3,862
F05	4	4	120ISI	2,632
F05	5	4	120ISI	10,791
F05	6	4	120ISI	6,23
F05	7	4	120ISI	9,683
F06	0	4	120ISI	0,767
F06	1	4	120ISI	0,869
F06	2	4	120ISI	0,889
F06	3	4	120ISI	0,786
F06	4	4	120ISI	1,099
F06	5	4	120ISI	0,972
F06	6	4	120ISI	0,835
F06	7	4	120ISI	0,884
F02	0	4	120ISI	9,888
F02	1	4	120ISI	1,704
F02	2	4	120ISI	2,422
F02	3	4	120ISI	4,888
F02	4	4	120ISI	2,197
F02	5	4	120ISI	1,748
F02	6	4	120ISI	8,281
F02	7	4	120ISI	1,67
F01	0	4	120ISI	1,191
F01	1	4	120ISI	1,016
F01	2	4	120ISI	0,937
F01	3	4	120ISI	0,742
F01	4	4	120ISI	0,937
F01	5	4	120ISI	1,406
F01	6	4	120ISI	0,82
F01	7	4	120ISI	1,147
M01	0	4	120ISI	1,133
M01	1	4	120ISI	0,913
M01	2	4	120ISI	0,972

M01	3	4	120ISI	0,864
M01	4	4	120ISI	0,977
M01	5	4	120ISI	0,962
M01	6	4	120ISI	0,869
M01	7	4	120ISI	0,957
F03	0	4	120ISI	10,576
F03	1	4	120ISI	7,773
F03	2	4	120ISI	7,319
F03	3	4	120ISI	2,222
F03	4	4	120ISI	3,506
F03	5	4	120ISI	5,347
F03	6	4	120ISI	11,069
F03	7	4	120ISI	3,267
M02	0	4	120ISI	0,884
M02	1	4	120ISI	0,835
M02	2	4	120ISI	2,563
M02	3	4	120ISI	1,006
M02	4	4	120ISI	1,719
M02	5	4	120ISI	1,821
M02	6	4	120ISI	1,206
M02	7	4	120ISI	0,889
F04	0	4	120ISI	1,865
F04	1	4	120ISI	1,636
F04	2	4	120ISI	2,061
F04	3	4	120ISI	1,812
F04	4	4	120ISI	1,782
F04	5	4	120ISI	1,934
F04	6	4	120ISI	1,812
F04	7	4	120ISI	1,963
F05	0	5	520ISI	1,162
F05	1	5	520ISI	9,634
F05	2	5	520ISI	10,273
F05	3	5	520ISI	10,557
F05	4	5	520ISI	10,2
F05	5	5	520ISI	10,444
F05	6	5	520ISI	11,636
F05	7	5	520ISI	9,668
F06	0	5	520ISI	0,82
F06	1	5	520ISI	1,147
F06	2	5	520ISI	9,219
F06	3	5	520ISI	1,133
F06	4	5	520ISI	0,908
F06	5	5	520ISI	1,738
F06	6	5	520ISI	1,377
F06	7	5	520ISI	3,901
F02	0	5	520ISI	8,706
F02	1	5	520ISI	7,847
F02	2	5	520ISI	5,132
F02	3	5	520ISI	7,153

F02	4	5	520ISI	1,87
F02	5	5	520ISI	2,622
F02	6	5	520ISI	4,355
F02	7	5	520ISI	3,804
F01	0	5	520ISI	1,958
F01	1	5	520ISI	3,94
F01	2	5	520ISI	2,642
F01	3	5	520ISI	2,246
F01	4	5	520ISI	1,265
F01	5	5	520ISI	1,318
F01	6	5	520ISI	0,918
F01	7	5	520ISI	2,114
M01	0	5	520ISI	0,967
M01	1	5	520ISI	1,084
M01	2	5	520ISI	0,835
M01	3	5	520ISI	0,942
M01	4	5	520ISI	0,937
M01	5	5	520ISI	0,962
M01	6	5	520ISI	0,771
M01	7	5	520ISI	0,962
F03	0	5	520ISI	10,405
F03	1	5	520ISI	11,372
F03	2	5	520ISI	11,313
F03	3	5	520ISI	8,296
F03	4	5	520ISI	9,58
F03	5	5	520ISI	2,627
F03	6	5	520ISI	7,441
F03	7	5	520ISI	9,541
M02	0	5	520ISI	1,821
M02	1	5	520ISI	1,68
M02	2	5	520ISI	7,5
M02	3	5	520ISI	5,723
M02	4	5	520ISI	1,978
M02	5	5	520ISI	8,364
M02	6	5	520ISI	2,725
M02	7	5	520ISI	2,368
F04	0	5	520ISI	2,549
F04	1	5	520ISI	2,368
F04	2	5	520ISI	5,454
F04	3	5	520ISI	2,974
F04	4	5	520ISI	2,144
F04	5	5	520ISI	3,364
F04	6	5	520ISI	2,334
F04	7	5	520ISI	2,695
F05	0	6	1020ISI	1,66
F05	1	6	1020ISI	1,279
F05	2	6	1020ISI	12,29
F05	3	6	1020ISI	8,652
F05	4	6	1020ISI	9,932

F05	5	6	1020ISI	9,253
F05	6	6	1020ISI	10,146
F05	7	6	1020ISI	10,312
F06	0	6	1020ISI	1,177
F06	1	6	1020ISI	1,899
F06	2	6	1020ISI	0,977
F06	3	6	1020ISI	4,224
F06	4	6	1020ISI	2,695
F06	5	6	1020ISI	0,664
F06	6	6	1020ISI	0,884
F06	7	6	1020ISI	1,587
F02	0	6	1020ISI	2,441
F02	1	6	1020ISI	4,053
F02	2	6	1020ISI	7,251
F02	3	6	1020ISI	4,878
F02	4	6	1020ISI	1,665
F02	5	6	1020ISI	8,799
F02	6	6	1020ISI	9,15
F02	7	6	1020ISI	2,842
F01	0	6	1020ISI	5,405
F01	1	6	1020ISI	2,593
F01	2	6	1020ISI	4,619
F01	3	6	1020ISI	3,745
F01	4	6	1020ISI	3,218
F01	5	6	1020ISI	1,416
F01	6	6	1020ISI	1,421
F01	7	6	1020ISI	2,769
M01	0	6	1020ISI	0,898
M01	1	6	1020ISI	0,864
M01	2	6	1020ISI	1,172
M01	3	6	1020ISI	1,025
M01	4	6	1020ISI	0,967
M01	5	6	1020ISI	1,25
M01	6	6	1020ISI	0,85
M01	7	6	1020ISI	0,894
F03	0	6	1020ISI	9,629
F03	1	6	1020ISI	9,795
F03	2	6	1020ISI	9,644
F03	3	6	1020ISI	8,027
F03	4	6	1020ISI	2,9
F03	5	6	1020ISI	8,574
F03	6	6	1020ISI	8,926
F03	7	6	1020ISI	10,562
M02	0	6	1020ISI	1,528
M02	1	6	1020ISI	2,944
M02	2	6	1020ISI	4,209
M02	3	6	1020ISI	5,586
M02	4	6	1020ISI	3,022
M02	5	6	1020ISI	3,315

M02	6	6	1020ISI	0,937
M02	7	6	1020ISI	0,889
F04	0	6	1020ISI	3,34
F04	1	6	1020ISI	3,696
F04	2	6	1020ISI	4,81
F04	3	6	1020ISI	9,668
F04	4	6	1020ISI	2,026
F04	5	6	1020ISI	2,632
F04	6	6	1020ISI	3,271
F04	7	6	1020ISI	3,398
