# Supplementary material for: Whole-Body Prepulse Inhibition Protocol to Test Sensorymotor Gating Mechanisms in Monkeys
Source: PLoS One. 2014 Aug 21;9(8):e105551. doi: 10.1371/journal.pone.0105551 (PMC4140807; doi:10.1371/journal.pone.0105551)
Supplement: Table S3 — Startle response of animals in Superior colliculus lesions test. (PDF) [file pone.0105551.s004.pdf]

Table S3. Startle response of animals in Superior colliculus lesions test.

Subject	Block	Trial	Test condition	PtoP
F01	0	0	115db	4,59
F01	1	0	115db	1,396
F01	2	0	115db	2,808
F01	3	0	115db	2,148
F01	4	0	115db	1,997
F01	5	0	115db	1,299
F01	6	0	115db	3,247
F01	7	0	115db	1,274
F01	8	0	115db	1,367
F01	9	0	115db	1,294
F06	0	0	115db	1,528
F06	1	0	115db	1,235
F06	2	0	115db	6,753
F06	3	0	115db	1,47
F06	4	0	115db	1,885
F06	5	0	115db	1,636
F06	6	0	115db	4,502
F06	7	0	115db	0,859
F06	8	0	115db	1,045
F06	9	0	115db	0,859
M01	0	0	115db	1,069
M01	1	0	115db	1,289
M01	2	0	115db	3,574
M01	3	0	115db	2,778
M01	4	0	115db	2,148
M01	5	0	115db	0,825
M01	6	0	115db	1,826
M01	7	0	115db	0,957
M01	8	0	115db	1,001
M01	9	0	115db	1,895
F03	0	0	115db	10,454
F03	1	0	115db	8,359
F03	2	0	115db	10,625
F03	3	0	115db	10,967
F03	4	0	115db	10,913
F03	5	0	115db	10,073
F03	6	0	115db	7,378
F03	7	0	115db	5,146
F03	8	0	115db	9,756
F03	9	0	115db	8,882
F02	0	0	115db	8,691
F02	1	0	115db	7,949
F02	2	0	115db	5,591
F02	3	0	115db	4,111
F02	4	0	115db	6,372
F02	5	0	115db	4,033

F02	6	0	115db	4,478
F02	7	0	115db	9,751
F02	8	0	115db	8,555
F02	9	0	115db	4,077
F04	0	0	115db	5,947
F04	1	0	115db	2,856
F04	2	0	115db	9,712
F04	3	0	115db	3,784
F04	4	0	115db	8,408
F04	5	0	115db	2,524
F04	6	0	115db	4,541
F04	7	0	115db	9,351
F04	8	0	115db	2,485
F04	9	0	115db	2,539
F05	0	0	115db	3,872
F05	1	0	115db	1,44
F05	2	0	115db	9,756
F05	3	0	115db	1,357
F05	4	0	115db	10,264
F05	5	0	115db	9,287
F05	6	0	115db	10,825
F05	7	0	115db	6,06
F05	8	0	115db	5,879
F05	9	0	115db	10,205
M01	0	0	115db	1,069
M01	1	0	115db	1,289
M01	2	0	115db	3,574
M01	3	0	115db	2,778
M01	4	0	115db	2,148
M01	5	0	115db	0,825
M01	6	0	115db	1,826
M01	7	0	115db	0,957
M01	8	0	115db	1,001
M01	9	0	115db	1,895
M02	0	0	115db	1,65
M02	1	0	115db	4,014
M02	2	0	115db	6,343
M02	3	0	115db	8,975
M02	4	0	115db	0,874
M02	5	0	115db	6,46
M02	6	0	115db	5,327
M02	7	0	115db	4,404
M02	8	0	115db	0,889
M02	9	0	115db	0,957
SC01	0	0	115db	4,36
SC01	1	0	115db	2,163
SC01	2	0	115db	1,646
SC01	3	0	115db	1,738
SC01	4	0	115db	2,422

SC01	5	0	115db	1,274
SC01	6	0	115db	1,357
SC01	7	0	115db	3,901
SC01	8	0	115db	1,294
SC01	9	0	115db	1,084
SC02	0	0	115db	2,192
SC02	1	0	115db	1,118
SC02	2	0	115db	0,933
SC02	3	0	115db	1,318
SC02	4	0	115db	1,084
SC02	5	0	115db	1,514
SC02	6	0	115db	1,387
SC02	7	0	115db	1,021
SC02	8	0	115db	2,354
SC02	9	0	115db	1,089
S01	0	0	115db	1,567
S01	1	0	115db	1,362
S01	2	0	115db	1,509
S01	3	0	115db	1,362
S01	4	0	115db	1,284
S01	5	0	115db	1,226
S01	6	0	115db	1,274
S01	7	0	115db	1,372
S01	8	0	115db	3,672
S01	9	0	115db	1,484
S02	0	0	115db	3,818
S02	1	0	115db	8,237
S02	2	0	115db	6,348
S02	3	0	115db	1,934
S02	4	0	115db	5,972
S02	5	0	115db	1,436
S02	6	0	115db	0,752
S02	7	0	115db	1,836
S02	8	0	115db	1,812
S02	9	0	115db	0,752
F01	0	1	80db	0,811
F01	1	1	80db	0,986
F01	2	1	80db	0,796
F01	3	1	80db	1,26
F01	4	1	80db	0,879
F01	5	1	80db	0,869
F01	6	1	80db	1,089
F01	7	1	80db	0,996
F01	8	1	80db	2,1
F01	9	1	80db	1,108
F06	0	1	80db	1,03
F06	1	1	80db	0,645
F06	2	1	80db	0,737
F06	3	1	80db	0,742

F06	4	1	80db	0,85
F06	5	1	80db	9,619
F06	6	1	80db	1,162
F06	7	1	80db	1,074
F06	8	1	80db	0,747
F06	9	1	80db	1,27
M01	0	1	80db	0,937
M01	1	1	80db	1,045
M01	2	1	80db	0,933
M01	3	1	80db	0,845
M01	4	1	80db	0,757
M01	5	1	80db	0,967
M01	6	1	80db	0,845
M01	7	1	80db	0,903
M01	8	1	80db	0,845
M01	9	1	80db	0,957
F03	0	1	80db	0,894
F03	1	1	80db	1,201
F03	2	1	80db	0,981
F03	3	1	80db	1,035
F03	4	1	80db	0,972
F03	5	1	80db	0,869
F03	6	1	80db	8,774
F03	7	1	80db	9,883
F03	8	1	80db	9,771
F03	9	1	80db	7,261
F02	0	1	80db	1,128
F02	1	1	80db	1,172
F02	2	1	80db	1,196
F02	3	1	80db	1,099
F02	4	1	80db	1,323
F02	5	1	80db	1,343
F02	6	1	80db	3,584
F02	7	1	80db	4,424
F02	8	1	80db	8,096
F02	9	1	80db	3,545
F04	0	1	80db	1,719
F04	1	1	80db	1,685
F04	2	1	80db	1,777
F04	3	1	80db	1,523
F04	4	1	80db	1,689
F04	5	1	80db	1,665
F04	6	1	80db	7,773
F04	7	1	80db	5,674
F04	8	1	80db	5,195
F04	9	1	80db	6,763
F05	0	1	80db	0,952
F05	1	1	80db	0,889
F05	2	1	80db	1,411

F05	3	1	80db	1,025
F05	4	1	80db	6,743
F05	5	1	80db	0,854
F05	6	1	80db	12,002
F05	7	1	80db	3,301
F05	8	1	80db	9,595
F05	9	1	80db	9,268
M01	0	1	80db	0,937
M01	1	1	80db	1,045
M01	2	1	80db	0,933
M01	3	1	80db	0,845
M01	4	1	80db	0,757
M01	5	1	80db	0,967
M01	6	1	80db	0,845
M01	7	1	80db	0,903
M01	8	1	80db	0,845
M01	9	1	80db	0,957
M02	0	1	80db	0,811
M02	1	1	80db	0,605
M02	2	1	80db	0,728
M02	3	1	80db	0,747
M02	4	1	80db	0,708
M02	5	1	80db	0,762
M02	6	1	80db	5,586
M02	7	1	80db	5,693
M02	8	1	80db	3,452
M02	9	1	80db	2,441
SC01	0	1	80db	0,947
SC01	1	1	80db	0,903
SC01	2	1	80db	1,021
SC01	3	1	80db	1,099
SC01	4	1	80db	1,191
SC01	5	1	80db	1,011
SC01	6	1	80db	1,045
SC01	7	1	80db	0,898
SC01	8	1	80db	0,933
SC01	9	1	80db	1,035
SC02	0	1	80db	0,947
SC02	1	1	80db	0,859
SC02	2	1	80db	0,845
SC02	3	1	80db	0,918
SC02	4	1	80db	0,967
SC02	5	1	80db	0,947
SC02	6	1	80db	0,898
SC02	7	1	80db	1,128
SC02	8	1	80db	1,05
SC02	9	1	80db	1,079
S01	0	1	80db	1,104
S01	1	1	80db	1,333

S01	2	1	80db	1,235
S01	3	1	80db	1,23
S01	4	1	80db	1,377
S01	5	1	80db	1,245
S01	6	1	80db	1,133
S01	7	1	80db	1,465
S01	8	1	80db	1,328
S01	9	1	80db	1,108
S02	0	1	80db	0,791
S02	1	1	80db	0,801
S02	2	1	80db	0,874
S02	3	1	80db	0,684
S02	4	1	80db	0,82
S02	5	1	80db	0,928
S02	6	1	80db	0,889
S02	7	1	80db	0,811
S02	8	1	80db	0,874
S02	9	1	80db	0,811
F01	0	2	120ISI	1,191
F01	1	2	120ISI	1,016
F01	2	2	120ISI	0,937
F01	3	2	120ISI	0,742
F01	4	2	120ISI	0,937
F01	5	2	120ISI	1,406
F01	6	2	120ISI	0,82
F01	7	2	120ISI	1,147
F01	8	2	120ISI	1,011
F01	9	2	120ISI	0,854
F06	0	2	120ISI	0,767
F06	1	2	120ISI	0,869
F06	2	2	120ISI	0,889
F06	3	2	120ISI	0,786
F06	4	2	120ISI	1,099
F06	5	2	120ISI	0,972
F06	6	2	120ISI	0,835
F06	7	2	120ISI	0,884
F06	8	2	120ISI	4,028
F06	9	2	120ISI	0,854
M01	0	2	120ISI	1,133
M01	1	2	120ISI	0,913
M01	2	2	120ISI	0,972
M01	3	2	120ISI	0,864
M01	4	2	120ISI	0,977
M01	5	2	120ISI	0,962
M01	6	2	120ISI	0,869
M01	7	2	120ISI	0,957
M01	8	2	120ISI	0,869
M01	9	2	120ISI	0,845
F03	0	2	120ISI	10,576

F03	1	2	120ISI	7,773
F03	2	2	120ISI	7,319
F03	3	2	120ISI	2,222
F03	4	2	120ISI	3,506
F03	5	2	120ISI	5,347
F03	6	2	120ISI	11,069
F03	7	2	120ISI	3,267
F03	8	2	120ISI	3,73
F03	9	2	120ISI	6,885
F02	0	2	120ISI	9,888
F02	1	2	120ISI	1,704
F02	2	2	120ISI	2,422
F02	3	2	120ISI	4,888
F02	4	2	120ISI	2,197
F02	5	2	120ISI	1,748
F02	6	2	120ISI	8,281
F02	7	2	120ISI	1,67
F02	8	2	120ISI	1,392
F02	9	2	120ISI	1,128
F04	0	2	120ISI	1,865
F04	1	2	120ISI	1,636
F04	2	2	120ISI	2,061
F04	3	2	120ISI	1,812
F04	4	2	120ISI	1,782
F04	5	2	120ISI	1,934
F04	6	2	120ISI	1,812
F04	7	2	120ISI	1,963
F04	8	2	120ISI	1,875
F04	9	2	120ISI	2,09
F05	0	2	120ISI	8,511
F05	1	2	120ISI	5,308
F05	2	2	120ISI	10,635
F05	3	2	120ISI	3,862
F05	4	2	120ISI	2,632
F05	5	2	120ISI	10,791
F05	6	2	120ISI	6,23
F05	7	2	120ISI	9,683
F05	8	2	120ISI	4,917
F05	9	2	120ISI	9,653
M01	0	2	120ISI	1,133
M01	1	2	120ISI	0,913
M01	2	2	120ISI	0,972
M01	3	2	120ISI	0,864
M01	4	2	120ISI	0,977
M01	5	2	120ISI	0,962
M01	6	2	120ISI	0,869
M01	7	2	120ISI	0,957
M01	8	2	120ISI	0,869
M01	9	2	120ISI	0,845

M02	0	2	120ISI	0,884
M02	1	2	120ISI	0,835
M02	2	2	120ISI	2,563
M02	3	2	120ISI	1,006
M02	4	2	120ISI	1,719
M02	5	2	120ISI	1,821
M02	6	2	120ISI	1,206
M02	7	2	120ISI	0,889
M02	8	2	120ISI	0,962
M02	9	2	120ISI	0,879
SC01	0	2	120ISI	1,631
SC01	1	2	120ISI	1,06
SC01	2	2	120ISI	4,756
SC01	3	2	120ISI	1,338
SC01	4	2	120ISI	1,089
SC01	5	2	120ISI	1,704
SC01	6	2	120ISI	1,084
SC01	7	2	120ISI	1,67
SC01	8	2	120ISI	1,069
SC01	9	2	120ISI	2,187
SC02	0	2	120ISI	0,913
SC02	1	2	120ISI	0,937
SC02	2	2	120ISI	0,996
SC02	3	2	120ISI	0,937
SC02	4	2	120ISI	1,143
SC02	5	2	120ISI	0,996
SC02	6	2	120ISI	0,854
SC02	7	2	120ISI	1,133
SC02	8	2	120ISI	0,908
SC02	9	2	120ISI	0,894
S01	0	2	120ISI	1,147
S01	1	2	120ISI	1,099
S01	2	2	120ISI	1,26
S01	3	2	120ISI	2,168
S01	4	2	120ISI	1,304
S01	5	2	120ISI	1,182
S01	6	2	120ISI	1,196
S01	7	2	120ISI	1,162
S01	8	2	120ISI	1,255
S01	9	2	120ISI	1,104
S02	0	2	120ISI	1,162
S02	1	2	120ISI	1,177
S02	2	2	120ISI	1,792
S02	3	2	120ISI	1,489
S02	4	2	120ISI	0,801
S02	5	2	120ISI	0,781
S02	6	2	120ISI	0,928
S02	7	2	120ISI	0,981
S02	8	2	120ISI	0,889

S02	9	2	120ISI	0,796
